# Diabetes confers *in vitro* calcific potential on serum which associates with *in vivo* vascular calcification

**DOI:** 10.1042/CS20160882

**Published:** 2017-05-09

**Authors:** Ashish Patidar, Dhruv K. Singh, Shori Thakur, Peter Winocour, Ken Farrington, Anwar R. Baydoun

**Affiliations:** 1 School of Life and Medical Sciences, University of Hertfordshire, College Lane, Hatfield, Hertfordshire AL10 9AB, U.K.; 2 Renal Unit, Lister Hospital, Coreys Mill Lane, Stevenage SG1 4AB, U.K.; 3 Department of Diabetes and Endocrinology, QEII Hospital, Howlands, Welwyn Garden City, AL7 4HQ, U.K.

**Keywords:** matrix Gla protein, osteoprotegerin, serum calcific potential, Type 2 diabetes, vascular aortic smooth muscle cells, vascular calcification

## Abstract

Although vascular calcification (VC) is prevalent in Type 2 diabetes mellitus (T2DM), underlying mechanisms remain unclear. Neither is it known whether T2DM confers calcific potential (CP) on serum, enabling it to induce VC outside the disease milieu. We, therefore, investigated the CP of serum from controls and subjects with T2DM with and without *in vivo* VC*.* Samples from 20 healthy controls and 44 age- and sex-matched patients with T2DM with modification of diet in renal disease estimated glomerular filtration rate (MDRD-4 eGFR) > 60 ml·min^−1^ were analysed for CP using rat aortic smooth muscle cells *in vitro*. CT scans of femoral arteries identified individuals with *in vivo* calcification. Serum from subjects with T2DM revealed significantly greater CP than controls. This was further enhanced in the presence of *in vivo* VC. Addition of β-glycerophosphate (β-GP) plus CaCl_2_ increased the CP of T2DM serum but not of controls. Along with age, CP was an independent predictor of the presence of VC. In receiver operator curve (ROC) analysis, CP was a significant predictor of femoral arterial VC (C-statistic 0.70: *P*=0.009). The distribution of CP was bimodal around a cutoff of 100 nmoles of Ca^2+^ protein mg^−1^, with a higher proportion of Type 2 diabetes subjects with *in vivo* calcification (T2DM+) sera above the cutoff value. This group also showed elevated levels of osteoprotegerin (OPG) and matrix Gla protein (MGP). Diabetes confers CP on the serum which is enhanced by the presence of *in vivo* VC. The CP acquired may be dependent on levels of OPG and MGP. These findings may be clinically relevant for early identification of individuals at risk of VC and for informing therapeutic strategies.

## Clinical perspectives

• Studies were conducted to investigate whether diabetes confers a calcific potential (CP) to serum, enabling the latter to induce vascular calcification (VC). Should this be the case, serum could be exploited to develop diagnostic and prognostic tests to determine an individual's risk of developing VC and inform therapeutic strategies.
• Serum from T2DM showed significantly greater CP than controls and correlated well with the presence of VC *in vivo*. The CP determined exhibited bimodal distribution with a higher proportion of sera from T2DM subjects with *in vivo* calcification. The T2DM group also showed elevated levels of osteoprotegerin and matrix Gla protein.
• Diabetes conferred CP to serum which, along with age, was an independent predictor of the presence of *in vivo* VC. The findings could be exploited for early identification of individuals at risk of VC and may lead to the design of effective therapeutic strategies to intervene with this process.

## Introduction

The major cause of mortality in subjects with Type 2 diabetes mellitus (T2DM) is cardiovascular disease. Vasculopathy is an early and seemingly inevitable feature of T2DM. It is linked to enhanced oxidative stress and endothelial cell dysfunction which act as an initial trigger for the progression of atherosclerosis and vascular calcification (VC) [1]. The latter is widely acknowledged to be a biologically orchestrated process in which vascular aortic smooth muscle cells (VASMCs) differentiate into an osteo/chondrogenic phenotype [2]. This phenotypic switch has been implicated in the pathogenesis of various diseases, including diabetes, with detrimental clinical consequences. Although patients with T2DM have significantly more VC compared with the general population, much remains unknown of the pathophysiology of VC in this setting. The underlying mechanisms which mediate this process have not been fully defined [3,4]. It is also not clear whether circulating factors generated prior to, or as a consequence of disease manifestation, initiate or contribute to the progression of calcification within the vessel wall.

Clinically, VC is assessed by non-invasive quantification of calcification using X-ray, computed tomography (CT) scanning or MRI. However, scanning not only requires expensive instrumentation but also detects VC when already established [5–7]. Currently, we have no means of predicting an individual's risk of developing VC. We have previously demonstrated that serum from individuals with chronic kidney disease (CKD) induces calcification of VASMCs *in vitro.* This effect, which we have referred to as the calcific potential (CP) of serum, increases with advancing CKD [8] but more importantly identifies individuals at risk of calcification under conditions which favour this process. To further validate our model and demonstrate whether CP is selective to CKD or a common characteristic of disease states associated with VC, we have extended our studies by assessing the capacity of serum from patients with T2DM to cause calcification. These studies were aimed at determining whether serum from patients with T2DM has a greater *in vitro* CP than normal age- and sex-matched controls and whether this correlates with *in vivo* calcification assessed by CT scanning. Such a phenomenon may have significant clinical implications. The findings could be exploited to develop diagnostic and prognostic tests to determine an individual's risk of developing VC and inform therapeutic strategies.

## Research design and methods

### Ethical approval

Ethical approval was obtained from the Hertfordshire Research Ethics Committee (Protocol number: 10/H0311/28). The procedures followed were in accordance with the ethical standards of the responsible committee on human experimentation (institutional and national) and with the Helsinki Declaration of 1975, as revised in 2008. The study subjects gave written informed consent prior to recruitment.

### Patient selection

Forty-four age-matched T2DM subjects and 20 healthy controls were studied. All were Caucasians and had preserved renal function with modification of diet in renal disease estimated glomerular filtration rate (MDRD-4 eGFR) > 60 ml·min^−1^. All the T2DM subjects were on oral hypoglycaemic drugs such as sulphonylureas and/or metformin (30 patients) and pioglitazone or rosiglitazone (10 patients). Four patients were additionally on insulin.

### Determination of *in vivo* vascular calcification

A CT scan of 5 cm segments of the femoral artery was carried out in both the groups as previously described to determine *in vivo* calcification [1,9]. The degree of VC was expressed as the Agatston score for each artery, and the greater of the two was used in subsequent analysis. Four groups were defined depending on the presence or absence of VC and designated as control with *in vivo* calcification (C+) or control without *in vivo* calcification (C−), and subjects with diabetes with *in vivo* calcification (T2DM+) and without *in vivo* calcification (T2DM−).

### Biochemical analyses of blood samples

Blood samples were taken from all subjects and routine analyses performed which included full blood count, blood urea, glucose, glycosylated haemoglobin (HbA1c), serum creatinine, albumin, calcium, phosphate and lipid profiles. Other samples for specified analysis including parathyroid hormone (PTH), osteoprotegerin (OPG), receptor activator of nuclear factor kappa B ligand (RANKL) and matrix Gla protein (MGP) were isolated in Z serum sep clot activator VACUTTE® by centrifugation at 2000 ***g*** for 15 min at 4°C and stored at −80°C until analysis. Serum levels of PTH were measured using the Beckman Access® 2 immunoassay system (Beckman Coulter, High Wycombe, U.K.). Assays for OPG and RANKL were performed using the automated enzyme-linked immunosorbent assay (ELISA) analyzer, Triturus® (Grifols, Cambridge, U.K.). MGP was measured using the BIOMEDICA GRUPEE Kit (BI-20062). All analyses were performed in duplicate.

### Induction and quantification of calcification

Rat VASMCs were isolated and cultured from aortic strips as described previously [10]. All experiments were carried out using cells at passage 3 to 5 and at a confluence of 60 to 70% in 96-well plates. The CP of sera samples was determined by incubating cells with 10% serum in standard Dulbecco's Modified Eagle's Medium (DMEM) supplemented with penicillin (100 U·ml^−1^) and streptomycin (100 μg·ml^−1^). In parallel studies, CaCl_2_ (7 mM) and β-glycerophosphate (β-GP; 7 mM) (calcification buffer; CB) in combination with 10% serum were used. Cells were lysed after 5 days of incubation using lysis buffer (10 mM Tris/HCl (pH 7.4) and 10% (w/v) SDS. Calcification was assessed using the DICA-500 Ca^2+^ assay kit [8] and total cell protein was determined using the bicinchoninic acid (BCA) assay as described previously [10]. Once determined, calcium levels in lysates were normalized with total cell protein per well and the data presented as nmoles of Ca^2+^ mg of protein^−1^.

### Materials

Cell culture and other general biochemical reagents including CaCl_2_ and β-GP were purchased from Sigma–Aldrich (London, U.K.). Penicillin and streptomycin were from Fisher Scientific (Loughborough, U.K.) and foetal bovine serum (FBS) from Gibco (Scotland, U.K.). The BCA protein quantification kit was from Thermo Scientific (Hemel Hempstead, U.K.) and the DICA-500 Ca^2+^ assay kit was from Universal Biologicals (Cambridge, U.K.). Reagents for the assay of OPG, RANKL and MGP were obtained from Oxford Biosystems (Oxford, U.K.).

### Statistical analysis

Statistical Package for Social Sciences (SPSS) version 17 (SPSS Inc., Chicago, U.S.A.) was used for data analysis and presented either as means ± S.D. or as median (interquartile range, IQR) as appropriate. For continuous variables, the significance of differences between two groups was determined by independent *t*-test (for normally distributed data) and the Mann–Whitney *U* tests or Wilcoxon test (for non-normally distributed data). Either one-way ANOVA or the Kruskal–Wallis test was employed as appropriate to assess differences between multiple groups. Categorical data were analysed using *χ^2^* test. Correlations were computed either as Pearson's or Spearman's for parametric and non-parametric distributed data respectively. Logistic regression analysis and receiver operator curve (ROC) analysis were used to examine the relationships of CP and the presence of VC.

## Results

### Comparison of biochemical variables and calcification in diabetes and control groups

There was no difference between the diabetes and control groups with respect to mean age or gender ratios (Table 1). Both groups had preserved renal function with eGFR more than 60 ml·min^−1^·1.73 m^−2^, though urinary albumin/creatinine ratio (ACR) was higher in patients with diabetes and serum albumin was slightly lower. There were significant differences in lipid profiles which were generally better in patients with diabetes, presumably because they were on lipid lowering therapy. Diastolic BP was also lower in diabetes patients. Serum PTH, OPG and RANKL levels did not differ between the groups though MGP levels were higher in those with diabetes.

**Table 1:** Comparison of biochemical variables and of calcification in diabetes and control groups Normally distributed parameters are presented as means ± S.D.; non-normally distributed parameters are presented as medians (IQR). DBP, diastolic blood pressure; eGFR (ml·min^−1^·1.73m^−2^), estimated glomerular filtration rate calculated using modification of diet in renal disease (MDRD-4) formula; HDL, high-density lipoprotein; NS, non-significant; SBP, systolic blood pressure.

Variables	Controls	Diabetes	*P-value*
Number	20	44	–
Age (yrs)	63±6	64±12	NS
Gender (M:F)	9/11	21/23	NS
SBP (mmHg)	149±24	149±19	NS
DBP (mmHg)	87±9	80±11	=0.025
Pulse Pressure (mmHg)	55(24)	69(22)	=0.07
Creatinine (μmol/l)	72±17	79±29	NS
eGFR (MDRD 4)	93±23	91±23	NS
ACR (mg/mmol)	0(3.2)	4.3(26.5)	<0.001
Calcium (mmol/l)	2.49±0.13	2.46±0.10	NS
Phosphate (mmol/l)	1.02±0.15	1.05±0.15	NS
PTH (pmol/l)	2.9(2.4)	3.4(2.9)	NS
Albumin (g/l)	45.4±2.5	43.6±3.1	=0.02
CRP > 5 mg/l (%)	15	10	NS
Cholesterol (mmol/l)	5.8±1.0	3.9±0.9	<0.001
HDL (mmol/l)	1.5±0.3	1.3±0.3	=0.008
Cholesterol/HDL	3.9±0.8	3.1±0.7	<0.001
OPG (pmol/l)	4.0±1.5	4.3±1.4	NS
RANKL (pmol/l)	0(0.06)	0(0.07)	NS
MGP (ng/ml)	15.9±1.4	17.7±3.4	=0.020
HbA1c (g/dL)	5.7±0.5	7.8±1.4	<0.001
DCCT (%; mmol/mol)	39±5	62±15	
Calcification Potential	37(41)	73(76)	<0.001
Agatston Score	0(173)	144(875)	=0.025

**Figure 1:**
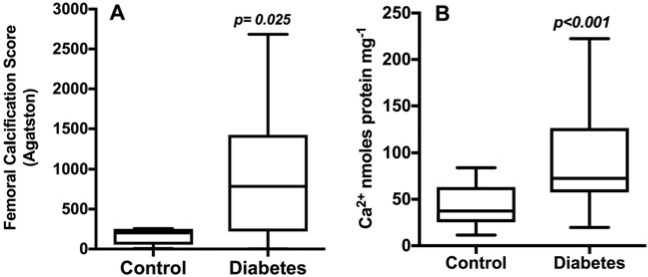
Agatston calcification scores (panel A) and *in vitro* CP of serum (panel B) from control and diabetes groups The data represent the medians and IQR. Figure (**B**) represents the data from three individual experiments with five replicates in each.

Agatston score was higher in patients with diabetes (Table 1 and Figure 1; panel A). The score correlated with age (rho=0.611, *p*<0.001), diastolic BP (rho=−0.402, *p*=0.002), pulse pressure (rho=0.547, *p*<0.001), serum creatinine (rho=0.303, *p*<0.01), urinary ACR (rho=0.315, *p*<0.01) and serum OPG (rho=0.439, *p*<0.001). There were no correlations with serum calcium, phosphate, PTH, RANKL and MGP.

Serum from subjects with diabetes had a higher median CP (73 (IQR 76)) than controls (37 (IQR 41): *p*<0.001) (Table 1 and Figure 1; panel B). The CP correlated positively with serum OPG (rho=0.360, *p*=0.004), MGP levels (rho=0.313, *p*=0.014) and pulse pressure (rho=0.327, *p*=0.011), but not with age, serum creatinine, eGFR, serum calcium, phosphate, albumin or PTH.

### Comparison of biochemical variables and calcification in diabetes and control groups stratified for the presence of *in vivo* calcification

The control and diabetes groups were further categorized on the basis of presence or absence of femoral calcification and divided into four groups: Group 1 (9 controls without *in vivo* calcification; C−), Group 2 (11 Controls with *in vivo* calcification; C+), Group 3 (17 DM without *in vivo* calcification; T2DM−) and Group 4 (27 DM with *in vivo* calcification; T2DM+).

Subjects with *in vivo* VC were older than their non-calcified counterparts in both the control and diabetes groups (Table 2). Diastolic blood pressure was lower in T2DM+ group compared with the C+ group. Renal function was not different between the groups, though urinary ACR was higher in those with diabetes and serum albumin levels were lower in T2DM+ than in C+. As previously mentioned, lipid profiles were generally better in those with diabetes. Serum calcium, phosphate, serum PTH and RANKL did not differ between groups. Serum OPG levels, however, were significantly higher in subjects with *in vivo* VC (either C+ or T2DM+) compared with subjects without *in vivo* VC. Also, T2DM+ had significantly higher levels of MGP than C+. There was no difference between HbA1c levels in T2DM− and T2DM+.

**Table 2:** Comparison of biochemical variables and calcification in diabetes and control groups stratified for the presence of *in vivo* calcification Normally distributed parameters are presented as means ± S.D.; non-normally distributed parameters are presented as medians (IQR). NS, non-significant.

Variables	C–	C+	T2DM–	T2DM+	C–vs C+*P*-value	C–vs T2DM–*P*-value	C+ vs T2DM+ *P*-value	T2DM–vs T2DM+ *P*-value
Number	9	11	17	27	NS	NS	NS	NS
Age (yrs.)	60±4	66±7	56±14	69±8	=0.038	NS	NS	=0.002
Gender (M:F)	4/5	5/6	7/10	14/13	NS	NS	NS	NS
SBP (mmHg)	140±11	159±29	146±15	150±21	=0.067	NS	NS	NS
DBP (mmHg)	87±9	88±10	84±10	77±12	NS	NS	0.028	=0.066
Pulse Pressure (mm/Hg)	52±11	72±28	61±15	73±19	=0.065	NS	NS	=0.075
Creatinine (μmol/l)	67±19	75±15	76±15	81±26	NS	NS	NS	NS
eGFR (MDRD 4)	98±28	90±20	99±21	87±23	NS	NS	NS	NS
ACR (mg/mmol)	0(8.8)	0(2.1)	3.8(12.9)	8.0(42.1)	NS	=0.058	<0.001	NS
Calcium(mmol/l)	2.43±0.12	2.53±0.13	2.46±0.11	2.46±0.09	NS	NS	NS	NS
Phosphate (mmol/l)	1.05±0.17	0.99±0.14	1.04±0.14	1.05±0.16	NS	NS	NS	NS
PTH (pmol/l)	3.1(1.4)	5.1(2.0)	3.4(1.8)	3.4(2.5)	0.08	NS	0.061	NS
Albumin (g/l)	44.8±2.8	45.8±2.3	44.5±2.1	43.1±3.6	NS	NS	=0.027	NS
Cholesterol (mmol/l)	6.4±0.9	5.4±0.9	3.8±0.9	3.9±0.9	=0.025	<0.001	<0.001	NS
HDL (mmol/l)	1.6±0.3	1.4±0.2	1.3±0.4	1.3±0.3	NS	=0.058	NS	NS
Cholesterol/HDL	4.1±1.0	3.8±0.6	3.1±0.8	3.1±0.7	NS	=0.014	=0.008	NS
OPG (pmol/l)	3.5±1.4	4.5±1.6	3.4±1.1	4.8±1.3	=0.013	NS	NS	=0.022
RANKL(pmol/l)	0(0.11)	0.0(0.02)	0.04(0.29)	0(0.03)	NS	NS	NS	NS
MGP (ng/ml)	16.4±1.6	15.3±1.2	17.7 ± 3.3	17.7±3.5	=0.085	NS	=0.036	NS
HbA1c(g/dL)	5.7±0.7	5.7±0.4	8.2±1.5	7.6±1.3				
DCCT (%; mmol/mol)	39±7	39±5	66±17	60±14	NS	<0.001	<0.001	NS
Calcification Potential	27(21)	52(65)	65(24)	83(82)	NS	=0.001	=0.027	=0.055
Agatston Score	–	86(246)	–	785(1423)			<0.001	

**Figure 2:**
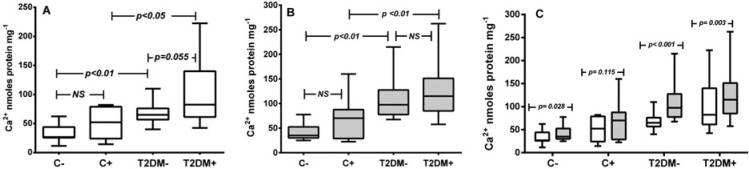
Effects of *in vivo* calcification on the *in vitro* CP of serum from control and diabetes groups The data shown in panel A are with serum alone, in panel B are for serum with CaCl_2_ (7 mM) plus β-GP (7 mM) and in panel C combined both (**A**) and (**B**). The data represent the medians and IQR from three individual experiments with five replicates in each.

The CP of sera from patients with VC (74 (IQR 85)) was higher than that of sera from those without VC (57 (IQR 44): *p*=0.009). The CP did not show statistically significant difference between C+ and C− but was significantly higher for T2DM+ when compared with these two groups (*p*<0.05; in both cases) and approached significance for sera from T2DM− (*P*=0.055) (Figure 2; panel A).

### Effect of addition of calcification buffer

The addition of CB revealed marked differences between CP of controls and of sera from subjects with diabetes (Figure 2; panel B). After the addition of CB, the CP was marginally enhanced in C− (*P*=0.028) and C+ (non-significant (NS), *P*=0.115), but significantly increased by comparison in T2DM− (*P*<0.001) and in T2DM+ (*P*=0.003; Figure 2; panel C), with that of the T2DM− being greater than in T2DM+ group.

### Relationship of *in vitro* CP and *in vivo* VC

The correlation between CP and Agatston score across all samples was weak (rho=0.209, *P*=0.097). In logistic regression analysis (Table 3), the independent determinants of the presence of femoral arterial VC were age and CP. The model accounted for 45% of the variation (Nagelkerke's *R*^2^=0.447). Adding other factors including the presence of diabetes, serum creatinine, MDRD-4 eGFR, urinary ACR, serum C-reactive protein (CRP), calcium, phosphate, PTH, RANKL, OPG and MGP did not improve the model significantly. In ROC analysis, CP was a significant predictor of the presence of femoral arterial VC (C-statistic 0.70: *P*=0.009). A cutoff of 100 nmoles of Ca^2+^ protein mg^−1^ predicted the presence of VC with a sensitivity of 33% and a specificity of 92%.

**Table 3:** Logistic regression analysis for determinants of the presence of femoral arterial calcification

						95% Confidence interval for Exp(*B*)	
	*B*	S.E.	Wald	*P*-value	Exp(*B*)	Lower	Upper
Age (years)	0.125	0.039	10.091	0.001	1.133	1.049	1.224
Calcification potential	0.020	0.009	5.097	0.024	1.020	1.003	1.037
Constant	−8.725	2.551	11.700	0.001	0.000		

### Distribution of calcific potential

**Figure 3:**
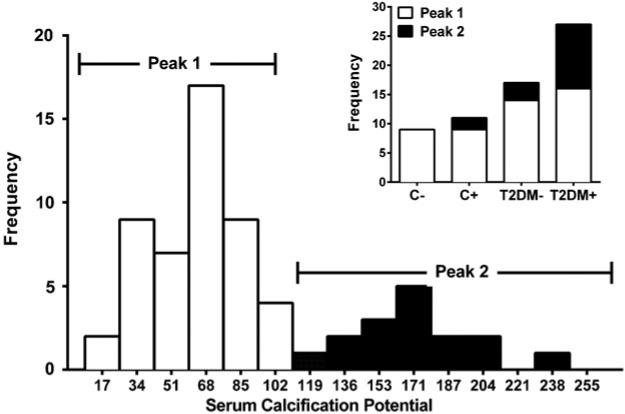
Frequency distribution of serum CP CP was plotted against the frequencies and divided further into two peaks, with peak 1 representing CP correlating with <100 nmoles Ca^2+^ protein mg^−1^ and peak 2 representing CP correlating with ≥100 nmoles Ca^2+^ protein mg^−1^. The inset shows numbers of subjects in the two peaks.

A plot of the distribution of CP was bimodal, consisting of 48 subjects with CP <100 and 16 subjects with CP ≥ 100 nmoles of Ca^2+^ protein mg^−1^ (Figure 3). Of the 16 subjects with the higher CP, 11 were T2DM+ (69%), 3 T2DM− (19%) and 2 C+ (12%) but none were C− (*P*=0.013 for T2DM+ compared with other groups combined). Moreover, 81% of subjects with CP values above the cutoff had *in vivo* VC compared to 52% with lower values.

Serum OPG was significantly higher in sera above the cutoff (5.2±1.3 compared with 3.9±1.3 pmol·l^−1^: *P*=0.005). Similarly, differences in MGP levels approached significance (18.3±3.5 compared with 16.7±2.8 ng·ml^−1^: *P*=0.078). In logistic regression analysis, both MGP and OPG were predictive of membership of the group above the cutoff (Nagelkerke's *R*^2^=0.269: *P*<0.001). Adding age, eGFR, serum levels of calcium, phosphate, CRP, the presence of diabetes and the presence of VC did not improve this model.

## Discussion

VC, a process of dynamic and pathological biomineralization of the vascular wall, involves VASMCs which may either calcify in the medial layer or migrate to the intima, resulting in medial and/or intimal calcification. Medial calcification is typically associated with diabetes [11], and until recently was thought to be related to the ageing process. It is now seen as a consequence of the smouldering inflammatory storm in patients with this condition, as in others such as CKD. It may also be due to the presence of promoting factors. Such factors may be present in the proximity of vascular cells and/or in the circulation, raising the possibility that sera from such settings may have the potential to induce VC outside the disease milieu. In this regard, we have previously reported on the *in vitro* CP of sera from CKD patients and further demonstrated this increases with declining renal function [8]. In the present study, we have extended these investigations and compared the CP of serum from controls and from two cohorts of patients with diabetes, those with and without *in vivo* VC to establish whether (i) serum from subjects with diabetes has *in vitro* CP and (ii) the *in vitro* CP correlates with the presence of VC *in vivo*. Both groups of subjects with diabetes had preserved renal function. The CP of sera from these two groups was compared with that of age- and sex-matched controls with no evidence of diabetes, CKD or any other major illness.

*In vivo* VC was detected in some patients with diabetes and, interestingly, in some subjects in the control group. The degree of VC in diabetes subjects was significantly higher than in controls, confirming that the presence of diabetes provides a favourable milieu for VC [1,12]. There was no significant difference in VC induced by serum from subjects with diabetes who were taking insulin compared with those not on this therapy, suggesting that the latter did not influence VC in our model. It is, however, worth noting that insulin has been reported to accelerate calcification but only at high doses [13]. We did not investigate the effects of insulin nor underlying mechanisms such as altered nitric oxide which may be involved in mediating its effects on calcification.

In both controls and diabetes groups, *in vivo* VC correlated with age and with OPG. There was also a trend for diabetics with VC to have lower diastolic pressures and increased pulse pressures, most likely a consequence of VC [14]. Renal function was identical across the groups though, unsurprisingly, the degree of microalbuminuria was greater in subjects with diabetes. Levels of CRP were similar between groups suggesting that inflammation may not be the main driver for calcification in this setting. Similarly, there were no differences in serum levels of calcium, phosphate and PTH. Serum OPG levels were elevated in the C+ and T2DM+ but not in the C− or T2DM− groups and this may be a factor in the increased CP of serum from these subjects. Serum MGP levels were slightly lower in controls with VC, and though higher in subjects with diabetes, they were not influenced by the presence of VC in this setting. Lipid profiles were generally better in patients with diabetes, probably because more of these subjects were taking lipid-lowering therapies.

The results obtained from our present studies have highlighted an interesting and novel property of serum from diabetes which has higher CP than controls. This is consistent with our initial observations on CKD sera [8]. It is also consistent with one other study demonstrating that culture of vascular smooth muscle cells in serum from subjects with diabetes and Charcot neuroarthropathy caused accelerated osteoblastic differentiation and mineralization indicative of VC [15]. These similarities in research findings suggest that CP is inherent in serum from disease settings associated with *in vivo* VC and highlight the value of cultured vascular smooth muscle cells as a model for the study of *in vitro* VC.

In addition to the findings reported above, we have also demonstrated that CP along with age is an independent predictor of the presence of VC. Moreover, 100 nmoles of Ca^2+^ protein mg^−1^ present in serum predict VC with a high specificity though with only moderate sensitivity. The CP determined exhibited bimodal distribution with a higher proportion of sera from subjects with *in vivo* calcification, particularly those with diabetes, falling within the higher CP group where Ca^2+^ levels were above the cutoff value of 100 nmoles of Ca^2+^ protein mg^−1^. Furthermore, levels of OPG and MGP within the high CP group were significantly higher than those with lower CP and appear predictive of the CP of serum. Thus, taken together, our data suggest that the CP of serum may be determined by the presence of diabetes, *in vivo* calcification and elevated OPG and MGP levels.

The CP of serum from subjects with diabetes and VC (T2DM+) was significantly greater than that in controls (C− and C+) and greater, though not significantly so, than that of T2DM− serum. These observations support the notion that the presence of diabetes confers CP on serum which is further enhanced in the presence of *in vivo* calcification. In controls, CP was higher in C+ than in C− but not significantly so. This is likely to be an effect of small sample size. There was also a significant age difference between the C+ and C− groups suggesting that age may contribute to the enhanced CP of serum from control subjects with *in vivo* calcification.

The underlying cellular mechanisms which may mediate CP have not been examined in detail. However, the changes we observed in OPG and MGP levels may be relevant since, along with RANKL, they are reported to regulate VC. Increased levels of serum OPG have been found in subjects with renal disease and diabetes and correlated with arterial stiffness [16–18]. Levels of OPG have also been reported to be elevated at sites of calcification within vessels, suggesting a role in the process [19]. Furthermore, Moreno et al. [16] demonstrated that a 1 pmol·l^−1^ elevation in OPG serum level amplified the risk of increased intimal medial thickness by 1.84, aortic calcification by 2.21 and peripheral artery disease by 4.02. These observations imply a critical role for OPG in the development of VC*.* Consistent with this are our observations that OPG levels were significantly higher in both the T2DM+ and C+ groups than their counterparts without VC (Table 2), and higher too in sera in the higher strata of the CP distribution (Figure 3). Elevated OPG expression may thus be a factor contributing to the higher CP of the serum.

Other mediators such as MGP present in sera from subjects with diabetes may also have a role. MGP exists in serum in the carboxylated (active) and/or uncarboxylated (inactive) form [20]. Carboxylated MGP inhibits the development of VC by negating the seeding of calcium phosphate crystals and inhibiting bone morphogenetic proteins [21], whilst uncarboxylated MGP may promote calcification. The ratio of uncarboxylated/carboxylated MGP levels and the expression of the γ-carboxylase enzyme have been correlated with medial artery calcification, aortic stenosis [22] and cardiovascular events [23]. Furthermore, reduced γ-carboxylation of MGP has been described in the context of the systemic inflammatory state associated with diabetes which would tend to enhance the concentration of uncarboxylated MGP [24], thus promoting VC in subjects with diabetes. We measured total MGP, but the higher CP of sera from subjects with diabetes might suggest a predominance of the uncarboxylated form. Further work is necessary in this area.

To determine whether CP could be potentiated within a calcifying environment, additional studies were carried out in which cells were incubated with serum in the presence of a CB as previously described using predetermined concentrations of CaCl_2_ and β-GP which induced VC but did not affect cell viability [8]. In the present studies, the CB did not cause any marked changes in the CP of control sera but significantly enhanced that from subjects with diabetes, particularly those without pre-existing VC. It is likely that serum from the latter group had already been primed *in vivo* and could, therefore, caused enhanced calcification under favourable conditions which promote the process even *in vitro*. In contrast, the limited increase in calcification seen with serum from the T2DM+ group may reflect a saturation effect of the potential to promote calcification in the presence of pre-existing calcification. What is important, however, is that sera from diabetes subjects exhibit enhanced CP which can be further augmented within a calcifying environment. The priming of the serum *in vivo* may be a consequence of changes in levels of promoters and/or inhibitors of calcification. This remains to be investigated.

Our study has some limitations. Firstly, the number of subjects was relatively small and may need to be increased in future studies to confirm significance, especially where these have been shown to be marginal. Secondly, the cross-sectional nature of the study precludes observations on the time course and in particular whether the CP of sera changes with disease progression and treatment. This is important given the potential use of *in vitro* and *in vivo* correlates of CP to estimate an individual's risk of calcification and to monitor the effects of therapy. Further work is necessary in this area and also on the mechanisms underlying these observations. This will allow us to infer whether the associations of OPG and MGP levels with diabetes and calcification are causal or just related to disease progression. Finally, our studies focused on Caucasians but a multi-ethnic study of atherosclerosis (MESA) has shown that Caucasians have the highest risk score for calcification followed by Chinese, Hispanics and African Americans [25]. We know of no studies that have compared the CP of serum in different ethnic groups. This will be addressed in our future studies.

In summary, using our model of VC, we have demonstrated that serum from subjects with diabetes possesses *in vitro* CP greater than that of controls and which is related to the degree of *in vivo* femoral artery calcification evident on CT scanning. That some patients with diabetes do not develop VC may provide an important mechanistic window to address in future studies. Future developments may allow the identification of biomarker profiles which may be useful in predicting risks of calcification in subjects with diabetes through a blood test rather than the use of expensive imaging techniques. In addition, it may also aid in monitoring responses to therapy, in exploring underlying mechanisms and in identifying potential novel therapeutic strategies for managing patients at risk.

## Abbreviations

ACR: albumin/creatinine ratio
BCA: bicinchoninic acid
BP: blood pressure
C+: control subjects with *in vivo* calcification
C−: control subjects without *in vivo* calcification
CB: calcification buffer
CKD: chronic kidney disease
CP: calcification or calcific potential
CRP: C-reactive protein
CT: computed tomography
HbA1c: glycosylated haemoglobin
IQR: interquartile range
MDRD-4 eGFR: modification of diet in renal disease estimated glomerular filtration rate
MGP: matrix Gla protein
OPG: osteoprotegerin
PTH: parathyroid hormone
RANKL: receptor activator of nuclear factor kappa B ligand
ROC: receiver operator curve
S.E.: standard error
T2DM: Type 2 diabetes mellitus
T2DM+: Type 2 diabetes subjects with *in vivo* calcification
T2DM−: Type 2 diabetes subjects without *in vivo* calcification
VASMC: vascular aortic smooth muscle cell
VC: vascular calcification
β-GP: β-glycerophosphate
